# Addressing treatment and care needs of older adults living with HIV who use drugs

**DOI:** 10.1002/jia2.25577

**Published:** 2020-08-26

**Authors:** Koharu Loulou Chayama, Cara Ng, Ryan McNeil

**Affiliations:** ^1^ British Columbia Centre on Substance Use Vancouver BC Canada; ^2^ Department of Experimental Medicine Faculty of Medicine University of British Columbia Vancouver BC Canada; ^3^ General Internal Medicine School of Medicine Yale University New Haven CT USA

**Keywords:** HIV, ageing, substance use, comorbidities, geriatric care, long‐term care, palliative care

## Abstract

**Introduction:**

Older adults living with HIV (OALHIV; ≥50 years) who use drugs face unique needs and challenges that compromise their health and wellbeing due to the structural and environmental barriers they experience, in addition to being disproportionately affected by comorbidities. Nevertheless, research on this population is limited and work is needed to tailor and optimize their care and services. The purpose of this commentary is to address the key research gaps pertaining to OALHIV who use drugs.

**Discussion:**

We identified four key research gaps specific to OALHIV who use drugs. Gap 1: Increased understanding of how older adults manage HIV alongside comorbidities in the context of substance use is critical to optimize their care management. Gap 2: More information on the geriatric characteristics of OALHIV who use drugs and the need and role of harm reduction in geriatric care is necessary for the provision of appropriate and effective care. Gap 3: Greater knowledge around the adoption of harm reduction and case manager approaches in various care facilities is essential to ensure equitable access to care for OALHIV who use drugs. Gap 4: Improved understanding of barriers to high‐quality palliative care among OALHIV who use drugs is important to enhance quality of life across their life course.

**Conclusions:**

Addressing the identified gaps in literature will lead to a more fulsome understanding of the issues encountered by OALHIV who use drugs and inform the development and implementation of strategies that address disparities at the intersection of HIV, substance use and ageing.

## 1 INTRODUCTION

Globally, close to 20% of people living with HIV (PLHIV) are over the age of 50 [1]. By 2020, the number of older adults living with HIV (OALHIV; ≥50 years) worldwide is expected to increase by 47% to 6.9 million [2]. This is due in large part to the success of antiretroviral therapy (ART) in increasing HIV survival rates and extending the lifespan of PLHIV [3]. This pattern has been most prominent in high‐income countries, where life expectancy of PLHIV is approaching that of the general population. However, similar improvements are now being seen in some parts of sub‐Saharan Africa [4, 5]. While much of the available literature on OALHIV is from high‐income countries, treatment and care that account for the changing demographics of HIV will be universally crucial as access to ART improves and leads to increases in life across the world.

OALHIV face myriad challenges that compromise their health and wellbeing, and these may be further magnified for those who use drugs [6]. At the same time, recent research on ageing has shown that, while ageing is associated with mortality, morbidity and decreased autonomy and functional independence, it is also associated with increased wellbeing and psychosocial functioning [7]. Support in the home and community are needed to achieve the positive outcomes associated with ageing [7]. This not only adds a layer of complexity but also informs research about optimal delivery of care and services for a population whose experiences are constituted at the intersection of HIV, substance use and ageing.

Substance use among OALHIV remains understudied as ageing among PLHIV is a relatively new phenomenon. While global estimates of the number and prevalence of PLHIV who use drugs has not been established, a recent systematic review estimated that globally, 17.8% (95% uncertainty interval 10.8 – 24.8 million) of people who inject drugs are living with HIV [8]. Unlike substance use patterns observed among the general population, previous research has suggested that substance use among PLHIV does not decline with age [9]. In one study from the United States (US), 10.5% of OALHIV were found to have used cocaine, 10.5% used crack cocaine, 6.3% used methamphetamines, 7.4% used heroin and 7.4% used prescription opioids without a prescription in the past 12 months [10]. Furthermore, close to a quarter of OALHIV were found to meet the DSM‐IV criteria for a substance use disorder [10]. Understanding drug use patterns of OALHIV is relevant to improving the health and wellbeing of this population, particularly as drug use has been found to be associated with sub‐optimal HIV treatment outcomes, including ART adherence [11]. Given that OALHIV who use drugs have unique needs and challenges, understanding current literature and addressing research gaps specific to this population (Table 1) may allow for more appropriate tailoring of care and services. Importantly, intersectional and equity‐oriented lens must be adopted in all future work addressing knowledge gaps for this population as OALHIV who use drugs occupy a wide range of social locations based on gender, sexuality, race, ethnicity, class, immigration status and other positionalities that intersect to shape experiences and outcomes in distinct but interrelated ways.

**Table 1:** Existing research and gaps regarding OALHIV who use drugs

What’s known
Unlike substance use patterns observed among the general population, substance use among PLHIV does not decline with age [9] Prevalence of substance use is disproportionately higher among OALHIV compared with those not living with HIV [12, 13] Many OALHIV meet criteria for living with substance use disorders [10] Substance use is a primary predictor of engagement in condomless sex and non‐adherence to medications among OALHIV as with their younger counterparts [14, 15] HIV infection and immunosuppression are associated with earlier onset of menopause in women living with HIV who use drugs [16]
What’s unknown
Management of comorbidities, including but not limited to mental illness (e.g. depression, anxiety), substance use disorders and cognitive impairment among OALHIV who use drugs [17] Management of geriatric conditions among OALHIV who use drugs Treatment regimens and polypharmacy in the context of substance use, HIV and ageing Integration of harm reduction in healthcare (e.g. palliative care, including end‐of‐life care) for OALHIV who use drugs Access barriers (e.g. stigma, discrimination) to long‐term care facilities for OALHIV who use drugs Psychosocial factors (e.g. social isolation, loneliness, social support) affecting the health of OALHIV who use drugs Clinician perceptions of people who use drugs and equitable delivery of pain care in OALHIV who use drugs

## 2 DISCUSSION

### Gap 1: comorbidities management in the context of HIV, substance use and ageing

PLHIV have a higher burden of comorbidities compared to those not living with HIV [18]. While ageing itself contributes to higher incidences of comorbidities, HIV can contribute to a range of co‐occurring health conditions, with the most prominent being metabolic complications, cardiovascular, renal, and liver disease, osteoporosis, chronic neurological complications and non‐AIDS malignancies [18, 19]. While these comorbidities can develop among PLHIV at all ages, appropriate management of comorbidities will become an increasingly common and important issue as they age [18]. Future work should pay close attention to comorbidities among OALHIV who use drugs as existing literature suggests that PLHIV who use drugs are at increased risk for medical and psychiatric comorbidities – including viral hepatitis (e.g. hepatitis B and C, particularly among people who inject drugs), tuberculosis, bacterial infections (e.g. staphylococcal and streptococcal infections among people who inject drugs), mental illness (e.g. depression, anxiety disorders), substance use disorders and chronic pain – resulting in greater morbidity and mortality than PLHIV who do not use drugs [20, 21, 22]. Risk factors in OALHIV who use drugs may vary greatly depending on an individual’s substance use patterns, comorbidities, current regimens and medical histories [23]. The question of how OALHIV who use drugs manage these comorbidities and, relatedly, polypharmacy, and how different substances might have unique interactions with specific comorbidities in this population remains largely unanswered in the literature. Furthermore, while many studies have reported adverse impacts of substance use on non‐adherence to medications, including ART, among PLHIV, knowledge remains limited on the mechanisms linking substance use with medication and treatment regimens among OALHIV [7, 24, 25]. Research that explores how older adults manage HIV alongside other co‐occurring health conditions in the context of substance use is warranted.

### Gap 2: geriatric care for OALHIV who use drugs

Geriatric conditions, such as risk of fall, delirium, vision impairment and mobility challenges, are associated with a decline in functional health and contribute to increased use of acute care services among older populations [26, 27]. Addressing geriatric conditions is a critical part of improving the care of older adults [26]. OALHIV bear risk factors for geriatric syndromes, including multimorbidity, chronic inflammation and psychosocial factors such as social isolation [12, 28, 29, 30]. Among OALHIV, including those with suppressed viral loads, frailty, cognitive impairment and difficulty with instrumental activities of daily living (e.g. medication management) are particularly common geriatric conditions [31]. Recognizing and treating geriatric conditions may be critical for OALHIV who use drugs as substance use‐related comorbidities and health behaviours may lead to health issues that overlap with particular geriatric conditions such as incontinence and cognitive impairment; however, most of the research in this area remains focused on alcohol and must branch out to probe the impacts of illicit drug use on geriatric conditions in the ageing population [32, 33]. Furthermore, substance use itself contributes to increased risk for geriatric conditions including cognitive changes and unintentional injuries (e.g. falls) [32]. While there have been growing efforts to integrate geriatric principles emphasizing patient‐centred, goal‐oriented care into HIV care, little has been done to address the specific geriatric conditions that may be experienced disproportionately by OALHIV who use drugs compared to the general older adult population or the ways specific substances may interact with specific geriatric conditions [32, 34]. Furthermore, little is known about the need and scope for the integration of harm reduction in geriatric care for OALHIV and research is warranted in this area.

### Gap 3: substance use among OALHIV in care facilities

As PLHIV age, access to appropriate care facilities such as assisted living, supportive housing and long‐term care will become an increasingly important component of the continuum of health and social services – that is, services to enable older adults to receive support from others for daily basic activities [35]. Although approaches to care for older adults range globally, and are contingent on the ways that healthcare is organized within specific national and local contexts, OALHIV who use drugs nonetheless face distinct access barriers that warrant attention. PLHIV have difficulties accessing care facilities due to HIV‐related stigma [36], and this is even more pronounced for PLHIV who use drugs as they also encounter drug‐related stigma and discriminatory policies that exclude people who use drugs and/or have drug convictions from accessing critical resources [37]. For example some nursing homes in the US do not admit patients who are on treatment for opioid use disorder [38]. Existing literature on healthcare services delivery suggests that integrating harm reduction improves access to and engagement with care among PLHIV who use drugs, and emphasizes the need for providers in care facilities to expand their understanding of harm reduction and take anti‐stigma trainings [6, 39, 40]. Harm reduction as an important underpinning approach goes beyond disease prevention and treatment to ending stigma and harm to people who use drugs. It also involves care settings and providers facilitating access to harm reduction services in a non‐judgemental fashion [6]. Previous research on an integrated HIV care facility with a supervised injection site in Canada has shown the positive effects of adopting harm reduction for OALHIV who use drugs, including reduced risk of overdose [41]. Such interventions, in addition to interventions that ensure a safe supply of drugs (e.g. hydromorphone), are particularly critical against the backdrop of fentanyl overdose crisis in several countries including the US and Canada [42, 43, 44].

Since HIV transmission and substance use is inextricably linked to inequality, they are disproportionately more prevalent in racial, ethnic, gender and sexual minorities. These individuals encounter complex barriers beyond those defined by their HIV status and/or substance use [45, 46]. To ensure equitable access to care facilities for all OALHIV who use drugs, their relationships with existing care facilities and experiences of intersecting stigma and discrimination will need to be explored in future research. Furthermore, case studies of success in the context of addressing structural‐environmental barriers and inequities in care services accessed by this population are critical for the development of evidence‐based programmes and policies that serve to improve access for all OALHIV who use drugs. Such research must be undertaken alongside efforts to scale up existing programs for older adults to ensure that the specific needs of OALHIV who use drugs are not deprioritized.

Finally, due to the complex challenges facing people who use drugs with co‐morbidities, best practices suggest that facilities like long‐term care adopt a “case manager” approach to delivering holistic healthcare, integrating medical and behavioural treatment from practitioners across disciplines as well as connecting clients to relevant social services within their communities [6]. Research is needed to examine the feasibility, acceptability and effectiveness of adopting case manager approaches in various forms of care settings accessed by OALHIV who use drugs.

### Gap 4: palliative care needs of OALHIV who use drugs

Research on palliative care needs including end‐of‐life care and quality of care provided to PLHIV is underdeveloped [47]. The World Health Organization (WHO) has deemed that palliative care is an essential component of the comprehensive package of care for PLHIV. Nevertheless, existing research points to inequitable access to palliative care among PLHIV [47]. People who use drugs are frequently restricted from accessing palliative care due to abstinence‐based approaches adopted by many providers [48]. Given this, access to palliative care among OALHIV who use drugs must be an area of further study to ensure that efforts in palliative care such as end‐of‐life care do not exclude the unique needs and challenges faced by members of this already marginalized population. Specifically, studies should examine interventions, such as (1) the delivery of palliative care in home and community settings that may be deemed safer and more acceptable by OALHIV who use drugs than formal healthcare settings and (2) the integration of harm reduction strategies (e.g. supervised drug consumption services) into public or formal healthcare settings. Furthermore, research should address disparities in the quality of palliative care. While pain management is a principal component of palliative care, clinician barriers to pain control for PLHIV, such as concerns about the possible development of addiction to prescription analgesics, have been documented (49). Understanding how such factors may hinder the delivery of high‐quality palliative care among OALHIV who use drugs is needed to reduce unnecessary suffering and enhance quality of life across the life course, including end‐of‐life.

## 3 CONCLUSIONS

Few ageing‐related studies have focused on PLHIV who use drugs. With an increasingly ageing population, care and services must be tailored to meet their unique needs. Addressing the identified gaps in literature will lead to a more fulsome understanding of the issues encountered by OALHIV who use drugs and inform the development and implementation of strategies that address disparities at the intersection of HIV, substance use and ageing.

## COMPETING INTERESTS

The authors declare that there are no competing interests.

## AUTHORS’ CONTRIBUTIONS

KLC and CN conducted the literature searches and wrote the manuscript. RM reviewed the manuscript.
